# Editorial for the Special Issue “Nutritional Regulation on Gut Microbiota, 2nd Edition”

**DOI:** 10.3390/microorganisms14081663

**Published:** 2026-07-30

**Authors:** Garry X. Shen

**Affiliations:** Diabetes Research Group, Department of Internal Medicine, University of Manitoba, 835-715 McDermot Ave, Winnipeg, MB R3E 3P4, Canada; garry.shen@umanitoba.ca; Tel.: +1-204-789-3816; Fax: +1-204-789-3987

The gut microbiome plays a key role in maintaining normal health. Food intake provides nutrients required for the growth and homeostasis of the body, as well as gut microbes. Research on the nutritional regulation of the gut microbiota has rapidly grown internationally during the last twenty years. *Microorganisms* published the first edition of “Nutritional Regulation on Gut Microbiota” in 2023 [1]. Based on the success of the first edition, the journal organized a second edition of the Special Issue, featuring eight articles from scientists in five countries describing unique research in a wide range of species and topics [2].

Among the eight studies presented in the second edition of “Nutritional Regulation on Gut Microbiota”, four studies examine the gut microbiota in livestock and compare the impact of diverse types of forages or growth subtypes on growth performance, and their relationship with gut health. The remaining studies investigate the effects of dietary supplements, probiotics or traditional herbs on health-related conditions and their relationship with gut microbiota changes in animal models or human subjects.

Dedousi et al. [3] supplemented the diets of laying hens with dried olive pulp at two inclusion levels, which increased the fecal abundance of the beneficial bacterial genera *Megasphaera* and *Megamonas* in 59-week-old birds compared with hens fed a control diet. At 39 weeks of age, hens receiving the higher olive pulp dosage produced eggs with the darkest yolk color among all treatment groups. Additionally, the proportion of broken eggshells was reduced by 15–34% in the olive-pulp-fed groups relative to the control group. This was associated with significantly thick egg shells in hens receiving olive pulp supplementation. These findings suggest that dietary olive pulp can improve egg quality through the modulation of the intestinal microbiota.

Wu et al. [4] demonstrated that partially substituting corn and soybean meal with barley, wheat bran, and rapeseed significantly improved growth performance in Wenshan cattle. This was reflected in increases in chest circumference, body height, length, and average daily weight gain, alongside a lowering of blood lipid levels. Dietary inclusion of barley, wheat bran and rapeseed markedly altered the gut microbial community, increasing the relative abundances of *Firmicutes*, *Proteobacteria*, and *Bacteroidetes*, whereas the abundance of *Spirochaetes* was significantly reduced compared to host on control diet. The results suggest that optimizing feed ingredient composition can positively modulate the gut microbiota and enhance the growth of Wenshan cattle.

Xu et al. [5] investigated the effects of dietary fermented purslane on growth performance, immune responses, intestinal microbiota, and metabolic characteristics in weaned piglets. Supplementation with 0.2% fermented purslane significantly improved average daily gain during the first 14 days after weaning. The treatment also reduced the incidence of diarrhea and lowered serum concentrations of the pro-inflammatory cytokine IL-6. In addition, the feeding of fermented purslane increased antioxidant enzyme activity in the piglets. In addition, fermented purslane supplementation increased the relative abundance of fecal *Clostridium*_sensu_stricto_1, *Tyzzerella*, and *Prevotellaceae*_NK3B31_group, as well as butyrate production, and decreased the abundance of *Lactobacillus*, *Bacillus*, and *Subdoligranulum* compared with the control diet. The findings indicate that fermented purslane feeding enhances growth performance, and improves immune function and intestinal health in weaned piglets, through modulation of gut microbiota composition [5].

Li et al. [6] investigated why some Jinhua piglets are born with low birth weight by comparing standard-birth-weight (SG) piglets with low-birth-weight (LG) piglets. They examined organ development, colon structure, short-chain fatty acids (SCFAs), the gut microbiota, and gene expression. Analysis revealed that LG piglets had a lower liver index, suggesting impaired organ development; their colons showed deeper crypts, fewer goblet cells, and greater inflammatory cell infiltration, indicating compromised intestinal health. LG piglets had reduced SCFA concentrations, particularly butyrate and isobutyrate, which are important for gut health, energy metabolism, and immune function. In addition, many beneficial gut microbes were less abundant in LG piglets than in SG piglets. An altered microbiota was associated with lower SCFA production, potentially contributing to poorer energy metabolism, weakened immunity, and impaired glucose metabolism. In conclusion, the findings suggest that disruptions in the colonic microbiota of LG piglets may negatively affect SCFA production, metabolism, and immune function. In contrast, the beneficial microbes found in standard-birth-weight piglets may help protect against these adverse changes and support healthier development.

Gestational diabetes mellitus (GDM) is first diagnosed during mid-late pregnancy, and increases the birthweight of fetuses and the risks of delivery complications and diabetes in mothers and offspring [7]. Sugino et al. [8] investigated the maternal microbiome in women with GDM receiving a diet higher in complex carbohydrates (60% complex carbohydrates/25% fat/15% protein, *n* = 18) or a conventional GDM diet (40% carbohydrates/45% fat/15% protein, *n* = 16). The complex carbohydrate diet was associated with increases in trimethylamine N-oxide, indoxyl sulfate, and several triglycerides, while the conventional GDM diet was associated with increases in hippuric acid and betaine. The microbiome of participants receiving the complex carbohydrate diet was enriched with carbohydrate-metabolizing genes and beneficial taxa such as *Bifidobacterium adolescentis*, while the conventional GDM diet was associated with taxa related to inflammation, including antimicrobial resistance and lipopolysaccharide biosynthesis. The results suggest that the complex carbohydrate diet and conventional GDM diets produce specific microbial and metabolic responses during pregnancy, which are associated with distinct host responses related to metabolism and inflammation.

Zhu et al. [9] investigated the effects of the uric-acid-degrading bacterial strain *Priestia megaterium* ASC-1, isolated from pickled cabbage, in rats with hyperuricemia. After 15 days of oral administration, the strain successfully colonized the intestine and significantly reduced serum uric acid levels by 67.24%. In addition, ASC-1 improved gut microbiota disturbances caused by hyperuricemia. These findings indicate that *P. megaterium* ASC-1 may serve as a promising probiotic adjunct for the treatment of hyperuricemia.

Zheng et al. [10] evaluated the effects of Yupingfeng polysaccharide (YPF-P) on the immune system, antioxidant capacity and gut microbiota in chickens. Supplementation with 0, 1, 2, or 4 g/kg of YPF-P in a regular diet significantly increased the thymus index and levels of IgA, IgG, and IgM, and strengthened antioxidant capacity by enhancing antioxidant enzyme activity and reducing malondialdehyde levels. In addition, YPF-P supplementation altered gut microbial composition by increasing the relative abundance of several beneficial bacterial genera, including *Faecalibacterium*, *Megamonas*, *Bacteroides*, *Alistipes*, NK4A214_group, and *Enterococcus*. Overall, YPF-P improved growth performance through its positive effects on immunity, oxidative balance, and intestinal microbiota in chickens.

Zhao et al. [11] evaluated the effects of brown rice (BRR) and germinated brown rice (GBR) on mice fed a high-fat diet. Both BRR and GBR increased fecal levels of isobutyric acid, while GBR additionally increased valeric acid levels compared with white rice supplementation. Elevated SCFA levels were associated with improved metabolic outcomes, including lower blood glucose, reduced insulin resistance, improved lipid profiles, and decreased inflammation. Moreover, increased isobutyric acid levels were positively associated with the abundance of *Lactobacillus*. These findings indicate that BRR and GBR may exert beneficial antidiabetic and anti-inflammatory effects by enhancing SCFA production and modulating the gut microbiota in high-fat-diet-fed mice.

The findings of the studies in this Special Issue may help to optimize the feeding of livestock, and potentially improve the management of immunologic and metabolic disorders in humans. The publications have already attracted 50 citations and thousands of reads, reflecting the high interest of scientists and researchers in this field. The success of this Special Issue series suggests the rapid growth in interest in the nutritional regulation of the gut microbiota related to health research and the livestock industry. The continuation of research in this field is expected to yield significant health and economic benefits for society.
